# Cancer-associated fibroblasts: an emerging target of anti-cancer immunotherapy

**DOI:** 10.1186/s13045-019-0770-1

**Published:** 2019-08-28

**Authors:** Tongyan Liu, Chencheng Han, Siwei Wang, Panqi Fang, Zhifei Ma, Lin Xu, Rong Yin

**Affiliations:** 1 Department of Thoracic Surgery, Jiangsu Cancer Hospital & Jiangsu Institute of Cancer Research & The Affiliated Cancer Hospital of Nanjing Medical University, Jiangsu Key Laboratory of Molecular and Translational Cancer Research, Collaborative Innovation Center for Cancer Personalized Medicine, Nanjing, China; 2 The Fourth Clinical College of Nanjing Medical University, Nanjing, China; 3 Department of Scientific Research, Jiangsu Cancer Hospital & Jiangsu Institute of Cancer Research & The Affiliated Cancer Hospital of Nanjing Medical University, Jiangsu Key Laboratory of Molecular and Translational Cancer Research, Nanjing, China; 4 Jiangsu Biobank of Clinical Resources, Nanjing, 210009 China; 5 Department of Clinical Pharmacy, School of Basic Medical Sciences and Clinical Pharmacy, China Pharmaceutical University, Nanjing, 210009 China

**Keywords:** Cancer-associated fibroblasts, Heterogeneity, Immune suppression, Cancer immunotherapy

## Abstract

Among all the stromal cells that present in the tumor microenvironment, cancer-associated fibroblasts (CAFs) are one of the most abundant and critical components of the tumor mesenchyme, which not only provide physical support for tumor cells but also play a key role in promoting and retarding tumorigenesis in a context-dependent manner. CAFs have also been involved in the modulation of many components of the immune system, and recent studies have revealed their roles in immune evasion and poor responses to cancer immunotherapy. In this review, we describe our current understanding of the tumorigenic significance, origin, and heterogeneity of CAFs, as well as the roles of different CAFs subtypes in distinct immune cell types. More importantly, we highlight potential therapeutic strategies that target CAFs to unleash the immune system against the tumor.

## Background

The concept of the tumor microenvironment (TME) in the initiation and progression of a multitude of malignancies has been recognized over the past decade [1, 2]. The TME or stromal is a multicellular system composed of cells from mesenchymal, endothelial, and hematopoietic origins arranged in the extracellular matrix (ECM), which interact closely with tumor cells, contributing to tumorigenesis. The tumor-TME crosstalk regulates, either positively or negatively, cancer progression. While the TME of early-stage tumors confers anti-malignancy functions, some cancer cells can tolerate the suppression and, in turn, reprogram the TME into one exerting pro-malignancy functions [3]. Within the TME infrastructure, the secreted products of a variety of immune and non-immune cell types, such as cytokines and chemokines, and the different components such as metabolites, hypoxia, angiogenesis, ECM remodeling, interstitial pressure, and pH changes drive a chronic inflammatory, pro-angiogenic, and immunosuppressive intratumoral environment [4]. In the past decade, the TME has been admitted as a target-rich environment for developing novel anticancer agents [5].

One of the most dominant components in the tumor stroma is cancer-associated fibroblasts (CAFs), which are spindle-shaped cells that build up and remodel the extracellular matrix (ECM) structure [6]. Without question, CAFs have been extensively studied in vitro owing to their ease of isolation and inherent plasticity. However, the “CAF population” remains poorly defined in terms of their origin, subtypes, and biology due to a high heterogeneity and a lack of specific markers [7]. Recently, numerous studies have demonstrated that CAFs have emerged as important regulators of the anti-tumor immune response [8, 9].

Fibroblasts are generally quiescent and can be activated in a wound healing response, also known as myofibroblasts [6]. The long-held notion of tumor as “wounds that never heal” [10] indicates that CAFs could be targeted for cancer therapy. Numerous preclinical studies have indicated CAFs could be selected as an emerging target of anti-cancer immunotherapy [6–8, 10].

In this review, we summarize recent advances of CAF phenotypic heterogeneity and function diversity with a particular emphasis on the roles of different CAF subtypes in distinct immune cell types. We also highlight the potential therapeutic strategies targeting CAFs in the field of cancer immunotherapy.

## The significance and biological properties of CAFs

### The significance of CAFs in tumorigenesis

Although increasing evidence indicates that CAFs represent one of the most abundant cancer stromal cell types and contribute a lot in various malignant phenotypes, it is still necessary to fully evaluate the significance of CAFs in solid cancer malignance based on The Cancer Genomic Atlas (TCGA) dataset at first.

**Fig. 1:**
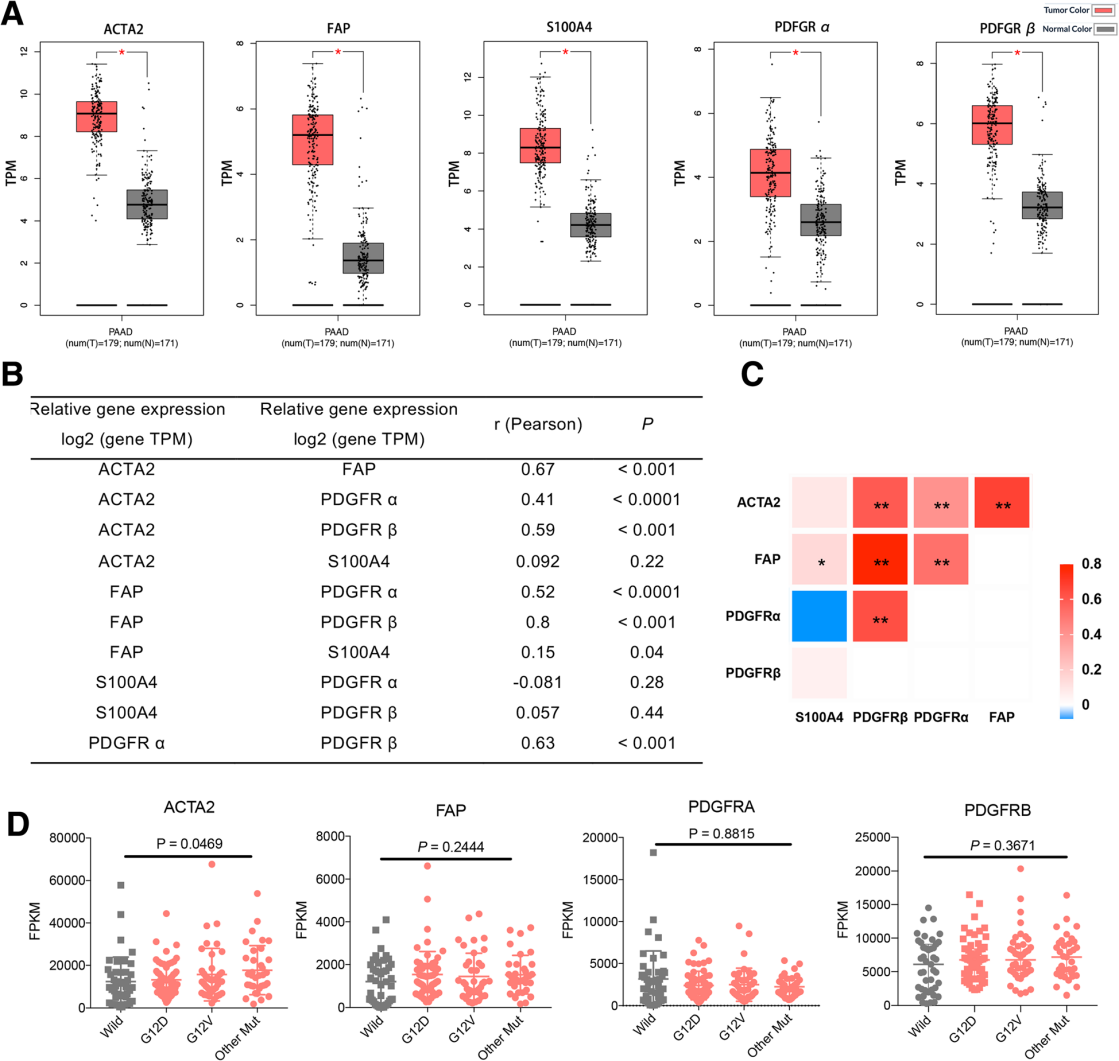
The tumorigenic significance of CAFs in PAAD. **a** The expression of CAF markers (ACTA2, FAP, PDGFRα, and PDGFRβ) was markedly overexpressed in pancreatic adenocarcinoma (PAAD) tissues compared with the paired normal tissues based on TCGA database. **b**, **c** The expression of ACTA2, FAP, and PDGFRα/β positively correlated with each other significantly. **d** Differential expression levels of ACTA2, FAP, PDGFRα and PDGFRβ among different KRAS status

Pancreatic adenocarcinoma (PAAD) remains one of the most common and lethal cancers in the world. Most importantly, since infiltrated CAFs in PAAD are most abundant in all solid cancers [11], we take PAAD as an example to evaluate the relationship between CAFs and cancer malignance phenotype based on TCGA database. As shown in Fig. 1a, we found that the expression of ACTA2, fibroblast activation protein (FAP), platelet-derived growth factor receptor-α/β (PDGFRα/β), and S100A4 (widely used as markers to define CAFs) were markedly overexpressed in PAAD tissues compared with the paired normal tissues (http://gepia.cancer-pku.cn). TCGA data analysis also showed that the expression of ACTA2, FAP, and PDGFRα/β in PAAD was positively correlated with each other significantly (Fig. 1b, c), except that S100A4 did not correlate with other markers. It is possibly attributed to the expression of S100A4 by quiescent or resting fibroblasts. It is reported that S100A4 may also serve as quiescent or resting fibroblast marker, while ACTA, FAP, PDGFRβ, and PDGFRα are predominantly expressed by CAFs [6].

Finally, although there was no direct evidence whether CAFs could induce somatic mutation and tumorigenesis, we attempted to detect the correlation between somatic mutations and CAF markers in TCGA data. Again the PAAD data demonstrated differential expression level of ACTA2 among different KRAS status (Fig. 1d, *P* = 0.0469). However, no significant results were found of other markers and a larger sample size is possibly needed. It is proposed that CFAs in TME may play a role in selecting tumor cells with specified driver mutation [12].

Together, the above data indicate that CAFs are a unique cell population significantly infiltrating in TME and contributing to the malignant phenotype and tumorigenesis.

### Biological properties: CAFs vs. normal fibroblasts

Fibroblasts in normal tissues are identified as resting mesenchymal cells embedded in physiological ECM. They can be activated to facilitate repair and regeneration during wound healing, tissue inflammation, and fibrosis. The corresponding processes in cancer development (“wound that never heals”) are tumor-promoting inflammation and tumor fibrosis [6]. As such, activated fibroblasts associated with cancer have been termed as CAFs [7]. Compared with quiescent fibroblasts, CAFs are generally larger, with indented nuclei and more cytoplasm branches under light microscopy [13]. In contrast to their normal counterparts, activated CAFs exhibit enhanced proliferative and migratory properties [7, 10]. Fibroblasts in normal tissue are commonly considered indolent with negligible metabolic and transcriptomic activity. However, CAFs are more metabolically active. The most unique feature of CAFs is their ECM production and synthetic phenotype [6]. Furthermore, CAFs can also produce many growth factors and proinflammatory cytokines, notably, transforming growth factor-β (TGF-β), vascular endothelial growth factor (VEGF), interleukin-6 (IL-6), and CXC-chemokine ligand (CXCL12), to promote angiogenesis and recruit immunosuppressive cells into the TME to assist in immune evasion [14, 15].

## Heterogeneity of CAFs

### Original heterogeneity

**Fig. 2:**
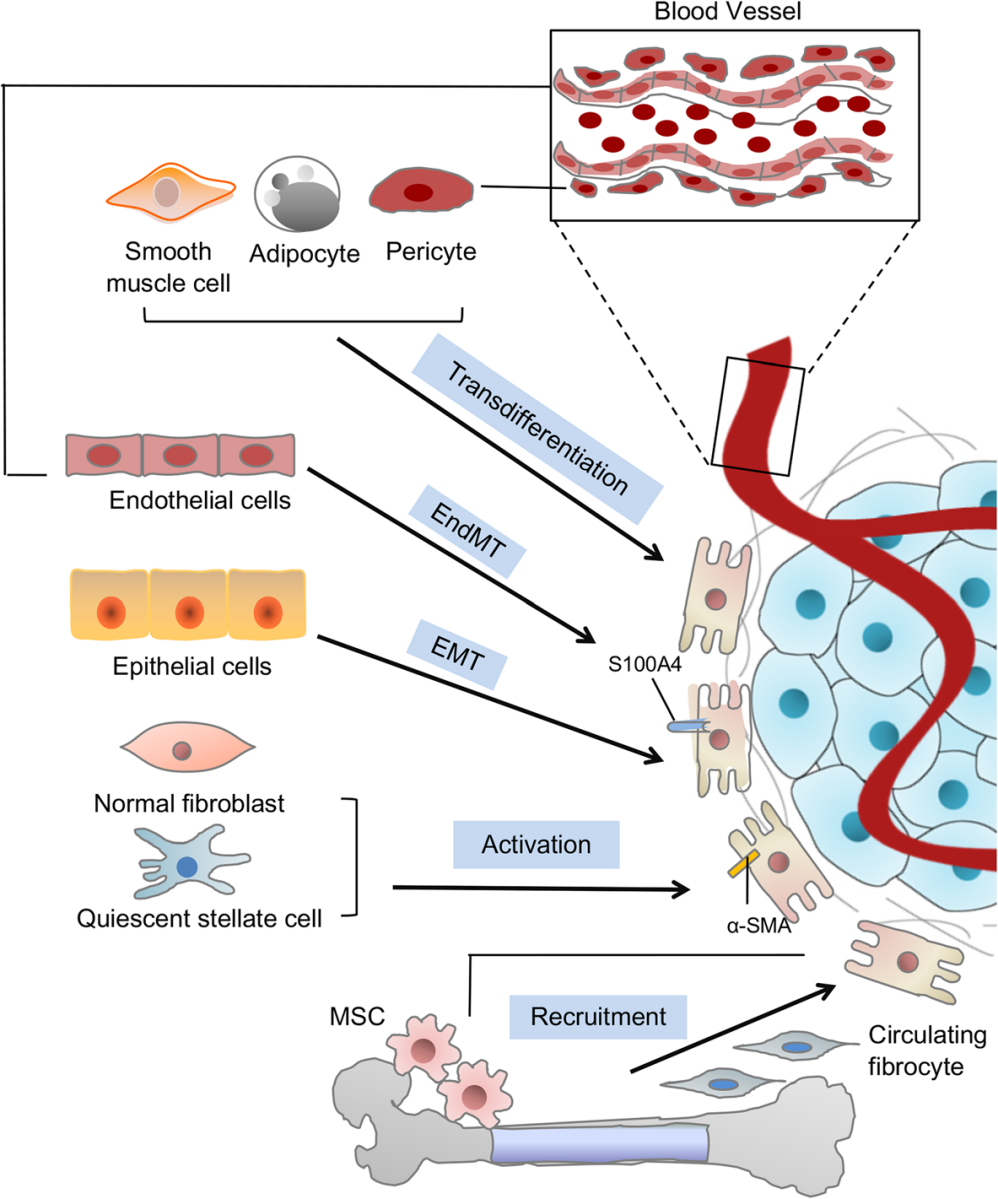
Potential cellular sources of CAFs. Cancer-associated fibroblasts (CAFs) can originate from diverse cell populations. Sources of CAFs include pre-existing resident fibroblasts and stellate cells (which become CAFs through activation), epithelial cells (via epithelial-to-mesenchymal transition, EMT), endothelial cells (via endothelial-to-mesenchymal transition, EndMT), mesenchymal stem cells (MSCs), and circulating fibrocytes (via recruitment), as well as pericytes, smooth muscle cells, and adipocytes (through transdifferentiation). ECM extracellular matrix; α-SMA α-smooth muscle actin; S100A4 fibroblast specific protein-1, also called FSP-1

Mounting evidence illustrates that CAFs are a heterogeneous population of cells [6]. Such heterogeneity might depend on the numerous cellular precursors of CAFs. CAFs can be recruited and activated from normal resident tissue fibroblasts [16, 17]. Similar to fibroblasts associated with wound healing [6, 7], this activation is largely depended on TME stimuli, such as local hypoxia, oxidative stress, and the growth factors released from the neighboring tumor cells and infiltrating immune cells. Fundamentally, TGF-β, epidermal growth factor (EGF), platelet-derived growth factor (PDGF), and fibroblast growth factor 2 (FGF2) are key regulators of fibroblast recruitment and activation [18, 19]. Moreover, immune cell-derived interleukin-1β (IL-1β) triggers nuclear factor-κB (NF-κB) activation in fibroblasts, involved in their education and proinflammatory secretome [20]. For example, resident fibroblast in the liver and pancreas, known as quiescent hepatic stellate cells (HSCs) and pancreatic stellate cells (PSCs), can acquire a myofibroblast-like phenotype, including α-smooth muscle actin (α-SMA) expression (which considered as CAFs in liver and pancreatic cancers, respectively) upon TGF-β and PDGF activation [21, 22]. In addition to the local sources, a portion of CAFs can transdifferentiate from non-fibroblastic lineage such as epithelial cells [23, 24], blood vessels [25], adipocytes, pericytes, and smooth muscle cells [26–28]. Generally, epithelial and endothelial cells undergo epithelial-to-mesenchymal transition (EMT) and endothelial-to-mesenchymal transition (EndMT), respectively, with an expression of S100A4 (fibroblast specific protein-1, also called FSP-1) and adopt a fibroblastic phenotype [29, 30]. Moreover, fibrocytes, a circulating mesenchymal cell population derived from monocyte precursors, may contribute to the pool of CAFs in TME, as occurs, for example in breast cancer [31]. Finally, CAFs may arise from typical bone-marrow-derived mesenchymal stem cells (BM-MSCs) in cancers such as glioma, breast, gastric, and pancreatic cancers [32–34]. Furthermore, tumor-associated MSCs (TA-MSCs) also originate from the naive MSCs and also have the potential to differentiate into CAFs, which warrants further mechanistic studies [10] (Fig. 2).

Compared with cancer cells, CAFs are generally considered more genetically stable [35]. Nevertheless, the cytotoxic challenge-induced mutations in the normal fibroblast may contribute to the generation of CAFs [7]. Furthermore, emerging data suggests that the irreversible conversion of fibroblast into CAFs might be driven by epigenetic alteration [36–38]. Collectively, the origins of CAFs are not fully elucidated. Lineage tracing methods could be used to identify the cellular origin of CAFs and monitor the development of CAFs during cancer evolution.

### Phenotypic heterogeneity

The various sources of activated fibroblasts lead to the phenotypic heterogeneity of CAFs, which can be manifested by diverse biological markers within the specific TME. Previous studies indicate that several markers, which are lower or not expressed by the normal counterparts, can be used to detect CAFs, such as α-SMA, S100A4, FAP, PDGFRα/β, tenascin-C, neuron glial antigen (NG2), desmin, CD90/THY1, and podoplanin (PDPN) [5, 7]. However, none of these markers is exclusively expressed by CAFs, most likely highlights the heterogeneity of CAFs. Among them, α-SMA is not only used to identify CAFs with a myofibroblast phenotype, but is also used as a general marker for vascular muscular cells and pericytes [39, 40]. S100A4, another well-known marker, is relatively specifically found on fibroblasts [41]. FAP is also found in a subset of CD45^+^ immune cells [42]. PDPN also identifies lymphatic endothelial cells [43]. A recent study has identified a new CAF subset (CD10^+^GRP77^+^) associated with cancer stemness and chemoresistance [44]. In another study, Mechta-Grigoriou et al. characterize four CAF subsets in breast and ovarian cancers with distinct properties by concomitant analysis of six fibroblast markers (FAP, αSMA, β1/CD29, S100A4, PDGFRβ, and caveolin1) [45, 46].

It is worth noting that further studies using single-cell RNA sequencing (scRNA-seq) have highlighted two CAF subsets in human colorectal tumors, with CAF-A cells expressing MMP2, DCN, and COLIA2 and CAF-B cells expressing ACTA2 (encoding α-SMA), TAGLN, and PDGFA [47]. A scRNA-seq study in patients with NSCLC shows lung tumors harbor five distinct fibroblast clusters. Remarkably, each of these fibroblast types expresses certain collagens or other extracellular matrix molecules, with for instance cluster 1 expressing COL10A1 and cluster 2 expressing COL4A1 [9]. Additionally, scRNA-seq on 768 CAFs derived from genetically engineered MMTY-PyMT mice bearing breast cancer revealed four subtypes of CAFs. Notably, PDGFRα is specifically expressed by subtype 2, while PDGFRβ is expressed by all cells with subtype 4 excluded. FAP, S100A4, and ACTA2 are generally expressed in four populations [48].

Currently, despite the diversity of CAF markers, defining a functional population of CAFs using cell surface markers stays challenging. Future studies could use scRNA-seq and in vivo models to interpret the heterogeneity of CAFs in the context of cellular origin, surface marker, RNA profiles, activation stages, and spatial distributions.

### Functional heterogeneity

Studies show that CAFs are composed of diverse functionally heterogeneous subpopulations that either promote or restrain cancer growth [6, 7, 10]. The pro-tumorigenic functions of CAFs have been investigated extensively based on in vitro and in vivo studies [49, 50]. For example, α-SMA^+^ CAFs utilize the CXC-chemokine ligand 12-CXC-chemokine receptor 4 (CXCL12-CXCR4) interaction to promote the proliferation of cancer stem cells [51]. Fundamentally, many other CAF-derived factors, such as matrix metallopeptidase 2 (MMP2), CXCL12, TGF-β, and IL-6, can promote the proliferation and invasion of cancer cells in various tumors [16]. However, the tumor-suppressive role of CAFs has been observed recently. For instance, the deletion of α-SMA^+^ myofibroblasts in pancreatic cancer suppresses immune surveillance by increasing CD4^+^Foxp3^+^ regulatory T cells (Tregs) in tumors [52]. Similarly, the deletion of fibroblast-rich desmoplastic stroma with sonic hedgehog inhibitor in pancreatic ductal adenocarcinoma increases the aggressive of tumors [53]. Interestingly, it is reported that breast TME harbors at least two CAF types based on CD146 expression. Specifically, CD146^−^ CAFs suppress estrogen receptor expression and the responsiveness of cancer cells to estrogen. However, CD146^+^ CAF can promote tamoxifen sensitivity to the luminal breast cancer cells [54].

Overall, CAFs have been involved in tumorigenesis, angiogenesis, metastasis, immunosuppression, drug resistance, maintenance of cancer stemness, ECM remodeling, and metabolic reprogramming [6, 48]. Nonetheless, for simplicity, we will elaborate on the following parts of this review on the tumor-promoting and immunosuppressive capabilities of CAFs and the potential immunotherapy strategies targeting CAFs.

## CAF-related anti-tumor immune response

### α-SMA^+^ CAF-mediated immunosuppressive in TME

**Fig. 3:**
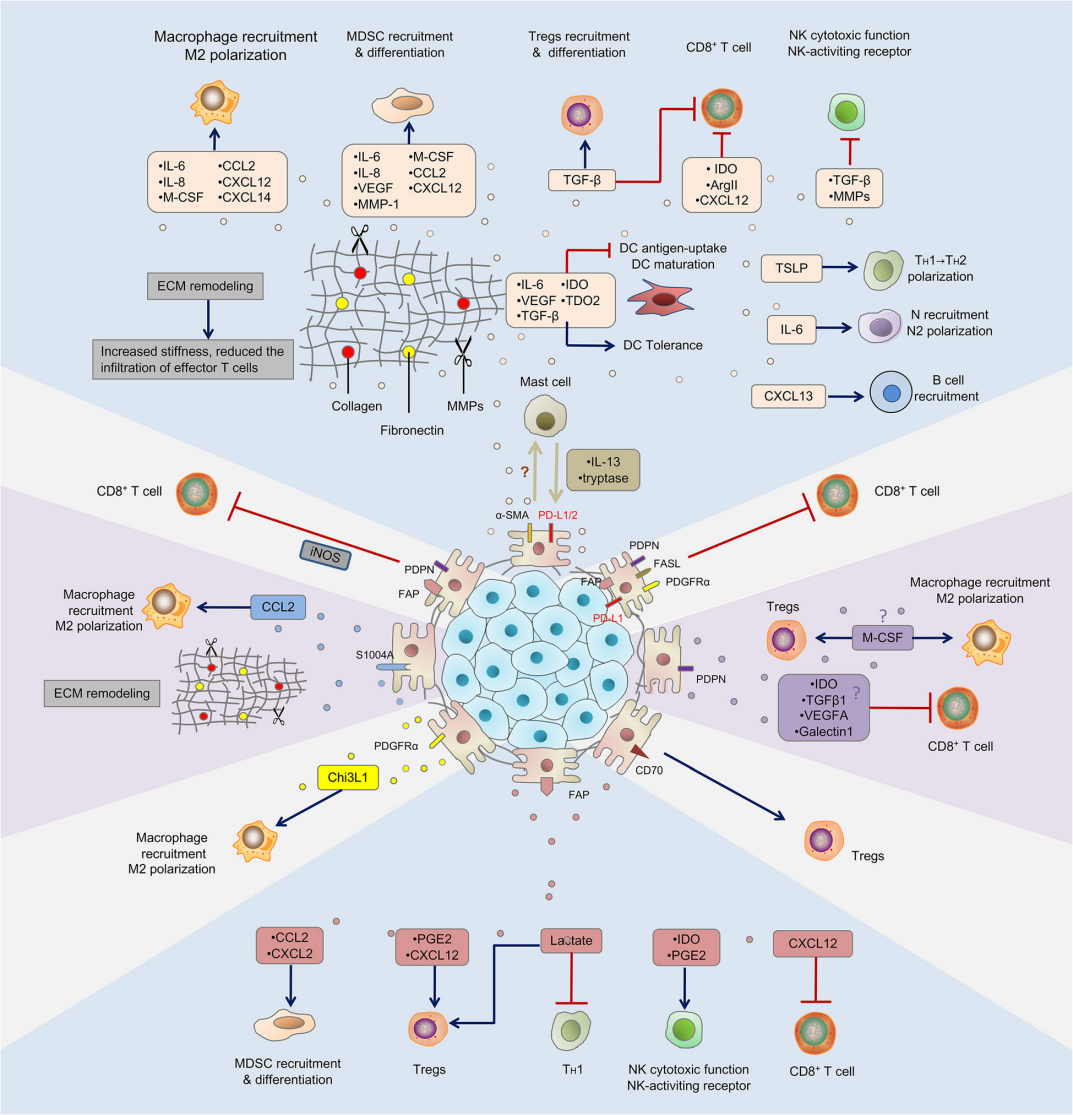
Immunosuppressive functions of different CAFs subtypes. Cancer-associated fibroblasts (CAFs) orchestrate an immunosuppressive tumor microenvironment. Different CAFs subtypes secrete numerous chemokines and cytokines, such as transforming growth factor-β (TGF-β), interleukin-6 (IL-6), interleukin-8 (IL-8), interleukin-13 (IL-13), CX-chemokine ligand 12 (CXCL12), CX-chemokine ligand 14 (CXCL14), and vascular endothelial growth factor A (VEGF), thereby inhibiting both the innate and adaptive anti-tumor immune response. Of note, some subpopulations express programmed cell death 1 ligand 1/2 (PD-L1/2), a target for immune checkpoint inhibitor. Metabolites or metabolic enzymes, such as indoleamine-2,3-dioxygenase (IDO), arginase (Arg), adenosine, and tryoptase produced by certain subtypes of CAFs favor the recruitment and differentiation of regulatory T cell (Tregs), mast cells, and tumor-associated macrophages (TAMs). Finally, CAFs can synthesize the extracellular matrix (ECM) components such as collagen, fibronectin, and matrix metalloproteinases (MMPs). Multiple CAF subtypes contribute to increased ECM stiffness, which in turn reduces the infiltration of effector T cells. MDSC myeloid-derived suppressor cell, DC dendritic cell, NK natural killer cells, T_H_ cells CD4^+^ helper lymphocytes, N neutrophils, FAP fibroblast activation protein, PDPN podoplanin, CCL2 chemokine ligand 2, M-CSF macrophage colony-stimulating factor, PDGFR platelet-derived growth factor, APC antigen-processing cell, FASL FAS ligand

α-SMA^+^ CAFs, also known as myofibroblasts, contribute to an immunosuppressive TME in various ways including paracrine and ECM remodeling (Fig. 3). Tumor-associated macrophages (TAMs) are the most abundant type of innate immune or inflammatory cell in close proximity to the CAF-populated areas, indicating a close association between these two cell types. In pancreatic cancer, α-SMA^+^ vimentin^+^ glial fibrillary acidic protein^+^ (GFAP), CAFs secret macrophage colony-stimulating factor 1 (M-CSF), IL-6, and CC-chemokine ligand 2 (CCL2) to promote monocyte recruitment encourage macrophage differentiation and M2 polarization [55]. The secretion of major cytokines, such as IL-6, IL-8, TGF-β, and IL-10 by α-SMA^+^ CAFs and α-SMA^+^ FAP^+^ CAFs, also actively increases the recruitment of monocytes and their differentiation into M2 phenotype [56, 57]. Reciprocally, TAMs with a M2 phenotype further activate CAFs and thereby promote tumor progression [58, 59]. In vitro studies show that α-SMA^+^ FAP^+^ CAFs educated MSCs (CAF-like MSCs) and can promote the invasiveness of TAMs [60]. Furthermore, the expression of both CAF markers (α-SMA, S100A4, and FAP) and M2 macrophages markers (CD163 and DC-SIGN) is correlated with the poor clinical outcome of squamous cell carcinoma and colorectal cancer patients [61, 62]. Moreover, α-SMA^+^ CAF-derived IL-6 can recruit neutrophils, activate signal transducer and activator of Janus kinase-programmed cell death ligand 1 (STAT3-PDL1) signaling cascade in neutrophils, therefore, contributing to immunosuppression in hepatocellular carcinoma [63]. Additionally, it has been reported that HSCs can activate mast cells; reciprocally, mast cell-derived IL-13 and tryptase can then active CAFs [64]. It is worth noting that activated mast cells not only increase tumor progression but also affects tumor immunity. For example, mast cell-derived IL-13 and adenosine might, respectively, promote M2 macrophage polarization and block the access of CD8^+^ T cells [65, 66]. Mast cells can also generate the infiltration of myeloid-derived suppressor cell (MDSCs) and Tregs in the TME [67]. However, how CAF-mast cell interaction is implicated to the tumor immunity is not fully elucidated and requires further investigation. Finally, as a major source of TGF-β, α-SMA^+^ CAFs can also regulate the activity of natural killer (NK) cells [5, 7]. Multiple studies have underscored the importance of TGF-β in suppressing NK cell activation and cytotoxic activity [68]. For example, TGF-β-induced miR-183 inhibits DAP12 transcription and decreases NK-activating receptor NKp30 and NK Group 2D (NKG2D) expression, resulting in restrained NK cytotoxicity [69]. TGF-β can also restrict the secretion of interferon- γ (IFN-γ) by NK cells, which is crucial for stimulating effector CD4^+^ T_H_1 cell-mediated anti-tumor reactions [68, 70]. A recent study in melanoma has also shown α-SMA^+^ CAF-derived MMP2 may cleave two ligands of the NK-activating receptor at the surface of tumor cells and consequently reduce the NKG2D-dependent cytotoxicity against melanoma tumor cells [8].

Besides the innate immunomodulatory functions, α-SMA^+^ CAFs might also interfere with the adaptive immune response at different levels. α-SMA^+^ CAF-derived TGF-β and IL-6 are implicated in restraining dendritic cells (DCs) function and maturation, disabling T cell activation and inducing T cell anergy [56, 70–72]. IL-6 signaling also redirects monocytes differentiated into macrophage rather than DCs [6, 73] and activates mast cells [6]. Furthermore, the tryptophan 2,3-dioxygenase (TDO2) and indoleamine-2,3-dioxygenase (IDO) released by α-SMA^+^ CAFs isolated from lung cancer enhance tryptophan degradation in kynurenines (Kyn) and consequently inhibits DCs differentiation and functions [8]. Finally, the VEGF derived from α-SMA^+^ CAFs suppresses DC generation and maturation [74, 75]. The role of α-SMA^+^ CAFs in modulating T cell activity and function has also been underscored. As described above, α-SMA^+^ CAFs are an important cellular source of TGF-β, which inhibits CD8^+^ T cell cytotoxic function by reducing the expression of perforin, granzymes A/B, FASL (FAS ligand), and IFN-γ[14, 76]. Activated PSCs (equivalent of CAFs in pancreatic cancer) secrete chemokines such as CXCL12 to sequester CD8^+^ T cells from accessing tumor cells [77]. Moreover, α-SMA^+^ FAP^+^ CAFs appear to inhibit the proliferation of CD8^+^ T cells and promote the recruitment of CD4^+^CD25^+^ T cells by secreting TGF-β and VEGF [78]. More importantly, α-SMA^+^ CAF-derived metabolic reprogramming factors, such as IDO1, Arg2, and galectin, are responsible for generating an immunosuppressive TME via inducing T cell anergy and inhibiting CD8^+^ T cell proliferation [79–81]. Additionally, in pancreatic cancer, α-SMA^+^ CAF-released thymic stromal lymphopoietin (TSLP) has been involved in T_H_2 cell polarization via myeloid DC conditioning [82]. Meanwhile, TGF-β, CXCL12, and VEGF secreted by α-SMA^+^ CAFs can favor the recruitment and differentiation of Tregs and T_H_17 cells [45, 46, 83–85]. In this regard, the coexistence of FoxP3^+^ Tregs and CAFs predicts poor outcome in lung adenocarcinoma [83]. It has been demonstrated that PSC-derived cytokines (such as IL-6, VEGF, and M-CSF) and chemokine (such as CXCL12 and CCL2) drive monocyte precursors toward an MDSC phenotype through STAT3 activation [8, 55]. Moreover, α-SMA^+^ CAFs in HCC attract monocytes to the tumor stroma by the secretion of CXCL12 and facilitate their differentiation into MDSCs in a IL-6-STAT3-dependent manner, thus contributing to the suppression of adaptive immune responses [86].

Interestingly, cultured α-SMA^+^ CAFs from colon tumor and melanoma carcinoma, as well as human fibroblast-specific 112 kDa surface molecule^+^(Thy1)α-SMA^+^FAP^+^ CAFs from lung cancer, have been reported to express immune checkpoint molecule programmed death 1 ligand 1/2(PDL-1 /2), which strongly induce T cell exhaustion [87–89]. Li et al. also report that α-SMA^+^ CAFs were positively correlated with PD-L1 expression by tumor cells in melanoma and colorectal carcinoma. Mechanistically, CX-chemokine ligand 5 (CXCL5) derived by α-SMA^+^ CAFs enhances the expression of PD-L1 in tumor cells by activating PI3K/AKT signaling pathway [90]. Finally, α-SMA^+^FAP^+^ CAFs were reported to respond to hypoxia and castration-caused tissue damage by promoting CX-chemokine ligand 13 (CXCL13) production, which aided B cells and other immunosuppressive cell trafficking to the TME that establish tumor progression [91, 92].

CAFs may also indirectly regulate the immune response through ECM remodeling [93, 94]. The modified ECM protein network serves as a physical barrier, blocking access of immune cells to the cancer cells [93, 95, 96]. For example, aligned fibronectin and collagen modified by α-SMA^+^ CAFs associate with poor cytotoxic T cell (CTL) infiltration [97–99]. Moreover, the extensive deposition of hyaluronic acid and collagen I, which can be highly secreted by α-SMA^+^ CAFs, improves TAMs infiltration [95]. However, the effect of ECM composition on Tregs, DCs, and neutrophils remains poorly understood.

Although α-SMA^+^ CAFs have potent immunosuppressive roles, α-SMA^+^ CAFs may also associate with the activation of tumor immune response. For example, myofibroblast-depleted mice with pancreatic ductal adenocarcinoma (PDAC) showed suppressed immune surveillance with increased CD4^+^ Foxp3^+^ Tregs infiltration [52]. α-SMA^+^S100A4^+^ CAFs have been reported to promote CD8^+^ T cells activation by fusion with DCs [100]. Such disparity possibly attributed to the existence of heterogeneous CAF subsets with α-SMA positive. Collectively, the α-SMA^+^ CAFs secretome might directly and indirectly regulate the anti-tumor immune response with many described and not yet elucidated manners.

### FAP^+^ CAF-mediated immunosuppressive in TME

Among the various CAF populations, the immunosuppressive role of FAP^+^ CAFs has been studied by different groups [15, 46, 101] (Fig. 3). FAP^+^ CAFs can induce monocyte recruitment and their differentiation into TAMs [8]. FAP^+^ CAFs inhibit the anti-tumor effect of M-CSF blockade by upregulating the infiltration of polymorphonuclear MDSCs in the TME [102]. Moreover, elevated FAP expression by CAFs can also recruit the circulating MDSCs into the tumor stroma through uPAR-FAK-DRC-JAK2-STAT3-CCL2 signaling pathway, thus resulting in immunosuppression in hepatic cancer [101]. Furthermore, in melanoma, hepatocellular, and colorectal carcinoma, FAP^+^ CAFs-derived prostaglandin (PGE2) and IDO can reduce the expression of NK-activating receptors, perforin and granzyme B, therefore, inhibit NK cell cytotoxicity and cytokine production [103]. As a principle source of CXCL12, FAP^+^ CAFs also use the CXCL12-CXCR4 interaction to inhibit the infiltration of T cells in PDAC and lung carcinoma bearing mice [15, 93, 104]. In this context, the blockade of CXCL12-CXCR4 axis improves sensitivity to checkpoint blockade therapy [15]. Consistent with this finding, Mechta-Grigoriou et al. have identified four subtypes of cancer-associated fibroblasts (CAF-S1-4) in human breast cancer and high-grade serous ovarian cancers by fluorescent-activated cell sorting, and found CAF-S1 subtype, characterized by elevated FAP expression, which is responsible for generating an immunosuppressive TME by accumulating CD4^+^CD25^+^ T cells and enhancing their differentiation to Tregs [45, 46]. CC-chemokine ligand 5 (CCL5) production by FAP^+^α-SMA^+^ CAFs in mammary carcinoma has appeared to preferentially recruit Tregs, owing to the highly expressed CC-chemokine receptor 1 (CCR1) by Tregs [5, 105]. A recent study also reveals that FAP^+^PDPN^+^ CAFs could regulate tumor-specific cytotoxic cell motility and localization through nitric oxide synthase (iNOS) [106]. Furthermore, in a prostate cancer model, FAP^+^ CAF-derived lactate is associated with increased Tregs and a shift in the polarization of CD4^+^ T cells from T_H_2 to T_H_1 phenotype, which depends on NF-kB signaling and FoxP3 expression [107]. In addition to the direct regulation of immune cell infiltration into the TME, FAP^+^ CAFs may also have a pivotal role in ECM remodeling; for example, FAP+ CAFs can produce TGF-β, VEGF, and multiple matrix processing enzymes [8, 10], indirectly disrupting the infiltration of cytotoxic T cells into the tumor nest.

Generally, FAP^+^ CAFs present a significant source of distinct chemokines and cytokines that can shape the immune landscape in the TME. Further research is required to elucidate how FAP^+^ CAFs participate in tumor immunosurveillance.

### Other subtypes of CAF-mediated regulation of tumor immunity

PDGFRα/β, S100A4, THY1 (CD90), and PDPN may also serve as marker sets to define CAFs. For example, Sugimoto et al. demonstrate that S100A4 identifies a unique subset of fibroblasts with minimal overlap with α-SMA, PDGFRβ, and chondroitin sulfate proteoglycan (NG2). Additionally, α-SMA, PDGFRβ, and NG2 could identify a mixed subtype of fibroblasts [108].

The immunomodulatory effects of PDGFRα/β^+^ CAFs and other subtypes have also been validated in multiple studies (Fig. 3). For instance, Chitinase-3-like-1 (Chi3L1), a secreted glycoprotein involved in chronic inflammatory and fibrotic disorders, has been linked to PDGFRα^+^ CAF-induced macrophage migration and their polarization into M2 phenotype [109]. S100A4^+^ CAF-derived CCL2 contributes to immune evasion by increasing the mobility and retention of macrophages [110]. Moreover, PDGFRβ^+^PDPN^+^FAP-α^+^ cells expressing FASL and PD-L2 induce the apoptosis of FAS-expressing CD8^+^ T cells and T cell anergy [111]. A recent study in stage I lung carcinoma has unveiled the role that PDPN^+^ CAFs have in attenuating anti-tumor immunity by decreasing the CD8/Foxp3 T cell ration, supporting monocyte recruitment and their differentiation into TAMs [112]. In vitro studies show CD70^+^ CAFs isolated from invasive colorectal cancer specimens stimulate the migration of Tregs. Meanwhile, the expression of CD70 on CAFs is proved to be an independent adverse prognostic marker for colorectal cancer [113]. Bone marrow-derived CAFs can also inhibit allogeneic T cell responses through IDO production [114]. Furthermore, collagen I^+^ CAFs increase TAMs trafficking to the stromal areas via hyaluronan-mediated ECM remodeling, thereby suppressing anti-tumor immunity [115]. Interestingly, S100A4-expressing CAFs may also increase immune surveillance ability through collagen production and encapsulation of carcinogens [41].

Although there has been increasing interest in cancer immunology, we are still beginning to understand the roles of CAF subtypes in tumor immunosurveillance. A brief summary of the features of representative CAF types including cell surface markers, cell origins, immune functions, and tumorigenic functions is listed in Table 1. Further studies are required to establish a deeper understanding of CAF heterogeneity and immunosurveillance.

**Table 1:** Commonly used CAF markers, their cellular origins and functions

Marker	Cell origins	Immune functions	Tumorigenic functions	Refs
α-SMA	Normal fibroblasts, quiescent stellate cells	Macrophage recruitment and M2 polarization, MDSCs and Tregs recruitment and differentiation, T cell anergy, NK cell inactivation, DCs tolerance and immaturation, TH_2_ and N2 polarization	Immuno-suppression, ECM remodeling, tumor cell proliferation, metabolic reprogramming, cancer stemness	[5, 7, 8, 49–90]
FAP	Normal fibroblasts, quiescent stellate cells	T cell anergy, NK cells inactivation, TH_2_ polarization, MDSCs and Tregs recruitment	Immuno-suppression, ECM remodeling, tumor progression and metastasis	[8, 10, 40, 41, 97–103]
S100A4	Epithelial cells, endothelial cells	Macrophage recruitment and M2 polarization	Immuno-suppression, ECM remodeling, carcinogenesis	[106]
PDGFRα/β	Normal fibroblasts, BMSCs, pericytes, vascular smooth muscular cells	T cell anergy and apoptosis	Immuno-suppression, tumor growth	[105]
PDPN	Epithelial cells	T cell anergy, macrophage recruitment and M2 polarization, Tregs recruitment	Immuno-suppression, tumor growth	[107, 108]
CD90		T cell exhaustion	Immuno-suppression, tumor cell migration	[83, 84]
Collagen I	Fibroblasts, vascular smooth muscular cells	Macrophage recruitment and M2 polarization	Immuno-suppression, ECM remodeling, angiogenesis	[111]

*α-SMA* α-smooth muscle actin. *FAP* fibroblast activation protein, *PDGFRα/β* platelet derived growth factor receptor-α/β, *PDPN* podoplanin, *MDSCs* myeloid-derived suppressor cell, *Tregs* regulatory T cell, *NK cells* natural killer cells, *N2* type2 neutrophils, *DC* dendritic cell, *ECM* extracellular matrix

## CAFs is a novel target in anti-tumor immunotherapy

**Fig. 4:**
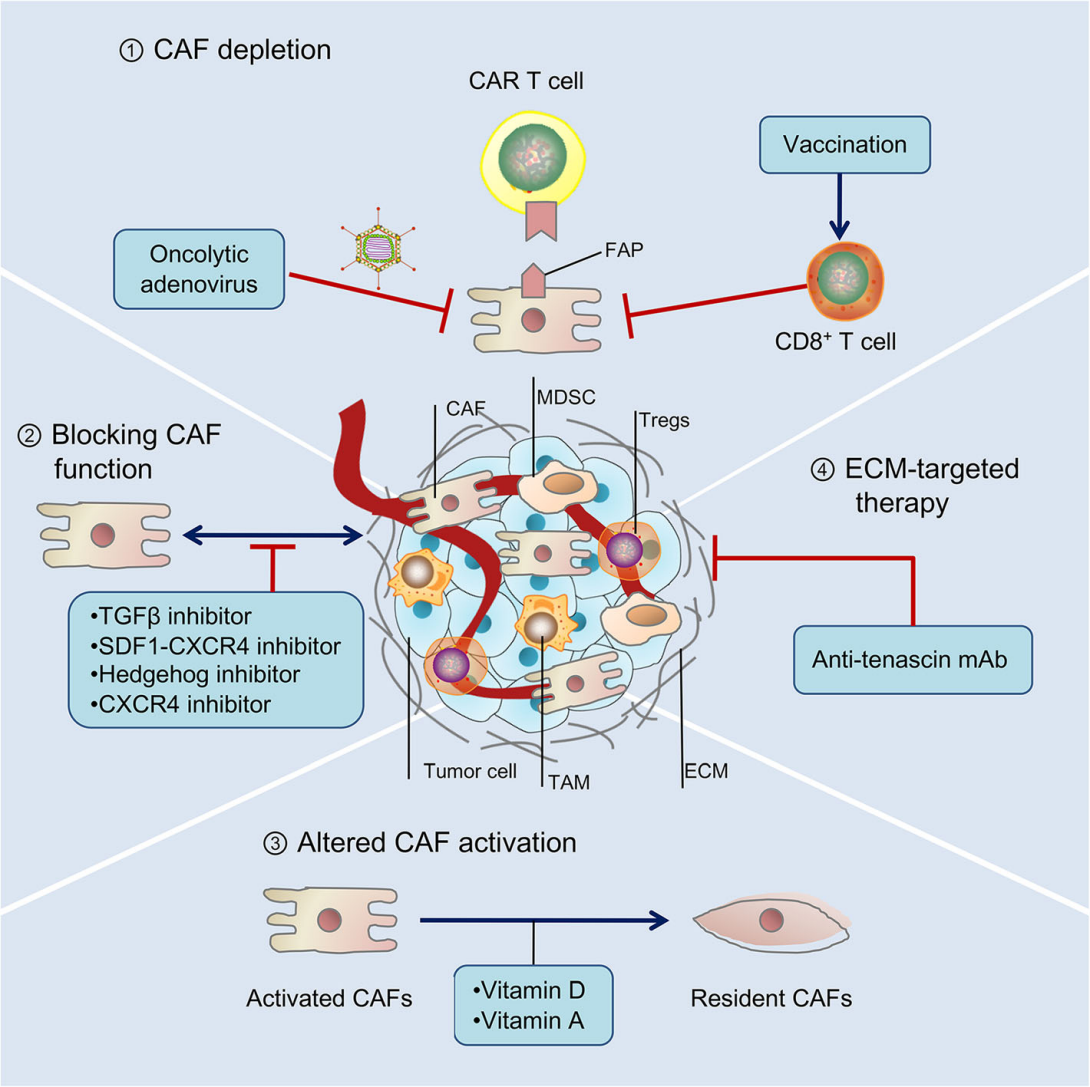
Immunotherapies that target CAFs. Four general approaches that target cancer-associated fibroblasts (CAFs) for cancer immunotherapy. ① Fibroblast activation protein^+^ (FAP^+^) CAFs can be directly eliminated by transgenic technologies, immunotherapies, and oncolytic adenovirus. ② Targeting the important signals and effectors of CAFs, such as CX-chemokine ligand 12-CX chemokine receptor 4 (CXCL12-CXCR4) interaction, Janus kinase-signal transducer and activator of transcription 3 (JAK-STAT3) pathway, transforming growth factor-β (TGF-β), and Hedgehog signaling pathway, can be used to inhibit the function of CAFs. ③ A reprogramming strategy such as vitamin A and vitamin D can be adopted to dedifferentiate activated CAFs to resident (normalized) fibroblasts. ④ CAF-derived extracellular matrix (ECM) proteins and associated signaling pathway can be targeted to induce stromal depletion. CAR chimeric antigen receptor, mAb monoclonal antibody, MDSC myeloid-derived suppressor cell, TAM tumor-associated macrophage, Treg cell regulatory T cell

The anti-tumor immunity that CAFs exert during cancer progression makes them promising therapeutic targets for cancer intervention. In the past few years, there has been considerable interest in developing “anti-CAF”-based immunotherapeutic approaches. Few of them have moved into the clinic; however, some CAF-related immunotherapy is in progress (Fig. 4).

Recently, anti-CAF therapies have been primarily focused on FAP [8]. Genetic deletion of FAP leads to a marked reduction in FAP^+^ CAF infiltration and rapid hypoxic necrosis of tumor and is associated with increased CD8^+^ T cells infiltration in Lewis lung carcinoma and PDAC models [116, 117]. Elimination of FAP^+^ CAFs by DNA vaccination and chimeric antigen receptor (CAR) T cells has emerged as important complements to other immunotherapeutic approaches. A pioneer study has shown oral administration of DNA-based FAP vaccine-induced CD8^+^ T cell-dependent killing of CAFs, which substantially increase the intratumoral uptake of chemotherapeutic drugs in multi-drug-resistant murine colon and breast carcinoma [118]. The development of a modified FAP DNA vaccine is capable of overcoming immune tolerance and inducing both CD8^+^ and CD4^+^ immune responses. The modified SynCon FAP DNA vaccine can synergize with other tumor antigen-specific vaccine therapies in tumor-bearing mice [104]. Of note, FAP-specific CAR T cell treatment in an immunocompetent mouse model has shown to boost host immunity. Similarly, co-introduction of anti-FAP and anti-tumor CAR T cells has also shown to enhance anti-tumor immunity in xenografted immunodeficient mouse models [119, 120]. Additionally, the adoptive transfer of FAP-specific CAR T cells can arrest pancreatic cancer growth with low immunogenicity and high desmoplasia [121]. Recently, oncolytic adenovirus with a FAP-targeting has displayed an improved anti-tumor immunity through endogenous T cell activation to attack FAP^+^ stromal cells in tumor-bearing mice models [122, 123]. However, it is important to note that BM-MSCs or skeletal muscles that express FAP may also be recognized and killed by FAP-reactive CAR T cells. As such, a contrasting result came from another study, in which adoptive transfer of FAP-reactive CAR-T cells not only had limited anti-tumor effects, but also had induced significant lethal toxicity and cachexia [116, 124]. These contrary results may attribute to the differential single-chain variable fragments (scFvs) constructed in the CARs; therefore, using FAP as a universal immunotherapy target should still be studied, albeit cautiously.

As discussed above, α-SMA identified at least the myofibroblast population of CAFs. In a mouse model of breast cancer, docetaxel conjugate nanoparticles that target α-SMA^+^ stromal suppressed metastases [125]. Selective depletion of myofibroblasts attenuated angiogenesis in spontaneous PDAC mouse models [126]. However, targeting α-SMA might increase the immunosuppressive CD3^+^Foxp3^+^ Tregs infiltrate in the TME, which ultimately led to aggressive tumor development [126].

Neither α-SMA nor FAP is exclusively expressed by CAFs, which substantially hinder the precision strategy of CAF-based therapy. In this scenario, targeting the cellular origins of CAFs may be another way to reduce CAF infiltration in the TME. A highly anticipated phase III clinical trial is ongoing to target the CAFs with endothelial cells precursors with bevacizumab [127].

In addition to the direct depletion of CAFs, it is also appealing to revert the CAF “state” by targeting the CAF activation pathways. In this context, CAF reprogramming by vitamin D and vitamin A, which reset the activated state of the pro-tumorigenic CAFs to a quiescent state, has attracted much attention in PDAC and colon cancer [128–130]. Administration of pleiotropic agent all-trans retinoic acid (ATRA) inhibits tumor-promoting signaling in activated PSCs, resulting in significantly increased infiltration of CD8^+^ T cells and improved therapeutic efficacy in PDAC models [131]. In a parallel study, the stimulation of the vitamin D receptor (VDR) successfully inactivates PSCs [132]. Notably, a phase II clinical trial is now underway with concomitant treatment with PD-1 inhibitor and vitamin D analog in PDAC [10].

Investigators are also targeting CAF-derived cytokines and chemokines in combination with immunotherapies in an attempt to improve anticancer efficiency [8, 10]. For example, a recent publication demonstrates that targeting the CXCL12-CXCR4 axis with AMD3100 (Plerixafor) reverses FAP^+^ CAF-mediated immunosuppression and synergizes with anti-PD-L1 immunotherapy in pancreatic cancer [15]. Similarly, other proteins released by CAFs, such as IL-6 and TGF-β, could also be targeted in order to improve the anti-tumor immune response [133]. For example, inhibitors of IL-6, IL-6 receptor, or Janus kinase (JAK) have already been approved by the US Food and Drug Administration for the treatment of myeloproliferative diseases and autoimmune disorders, with trials underway in cancer [134]. Novel agents that target IL-6 and its signaling pathway, including ROCKs and STAT3, have undergone clinical or preclinical trials in cancer [134]. Furthermore, TGF-β signaling in fibroblasts is shown to attenuate tumor response to anti-PD-L1 agent by contributing to T cell exclusion. Therapeutic co-administration of TGF-β-blocking and anti-PD-L1 antibodies inhibit TGF-β signaling in CAFs, facilitated T cell penetration into the tumor nest, and, therefore, provoke effective anti-tumor immunity and tumor regression [135, 136]. Tranilast (Rizaben), which suppresses fibroblast growth and TGF-β secretion, synergistically enhances the effect of dendritic cell-based vaccines in C57BL/6 mice with E-G7 lymphoma, LLC1 Lewis lung cancer, or B16F1 melanoma [137]. Notably, multiple phase I clinical trials of TGF-β-based immunotherapies are ongoing, highlighting the clinical importance of stroma-based immunotherapy [68]. Investigators are also using tenascin C inhibitor (^131^I-m81C6) or Hedgehog inhibitors in combination with immunotherapies and standard chemotherapies in order to ameliorate ECM stiffness to favor drug delivery [138, 139].

Overall, drugs that target CAFs have emerged as a critical complement to immunotherapies in multiple solid tumors. A brief summary of immunotherapeutic strategies that target CAFs in clinical and preclinical studies is given in Table 2. More specific molecular targets that alter CAF signals and effectors await further mechanistic and functional investigation.

**Table 2:** Immunotherapeutic strategies that target CAFs in clinical and/or preclinical studies

Drugs	Mechanisms	Combination therapy	Biological effects	Cancer models	Status	Refs
SynCon DNA vaccine	FAP deletion	Anti-tumor vaccine	Overcomes immune tolerance	Lung cancer	Preclinical	[100]
FAP-specific CAR T cell	FAP deletion	Anti-tumor CAR T cell	Enhances anti-tumor immunity	Lung cancer	Preclinical	[116]
Oncolytic adenovirus	FAP deletion	No	Enhances anti-tumor immunity	CRC	Preclinical	[118, 119]
ATAR (vitamin A analog)	Vitamin A storage and PSC deactivation	Gemcitabine	Enhances anti-tumor immunity; inhibits tumor cell growth	PDAC	Preclinical	[126]
Calcipotriol (vitamin D analog)	Vitamin D receptor activation and PSC deactivation	Anti-PD-1 immunotherapy	Reverses tumor immunosuppression	PDAC	Phase II	[10]
AMD3100	Blocks the CXCL12-CXCR4 interaction	Anti-PD-L1 immunotherapy	Reverses tumor immunosuppression	PDAC	Preclinical	[96]
^131^I-m81C6 (anti-tenascin mAb)	Radioimmunotherapy	No	Reverses tumor immunosuppression	Recurrent malignant glioma	Phase II	[135]
Ruxolitinib (JAK inhibitor)	JAK-STA3 pathway inhibition	Capecitabine	Inhibits tumor-promoting inflammation	Metastatic pancreatic cancer	Phase II	[129]
IPI-926	Hedgehog pathway inhibition	Gemcitabine	Reverses tumor immunosuppression	PDAC	Preclinical	[133]
NIS793 ABBV151	Blocking pan-TGF-β and GARP	Anti-PD-1 immunotherapy	Reverses tumor immunosuppression	Breast, lung, HCC, CRC, pancreatic and renal cancer	Phase I	[62]

*FAP* fibroblast activation protein, *CAR* chimeric antigen receptor, *ATRA* all-trans retinoic acid, *CXCL12* CXC-chemokine ligand 12, *CXCR4* CXC-chemokine receptor 4, *PD-1* programmed cell death-1, *PD-L1* programmed cell death 1 ligand, *JAK* Janus kinase, *STAT3* signal transducer and activator of transcription 3, *mAb* monoclonal antibody, *PSC* pancreatic stellate cell, *TGF-β* transforming growth factor-β, *GARP* glycoprotein A repetitions predominant protein, *PDAC* pancreatic ductal adenocarcinoma, *HCC* hepatocellular carcinoma, *CRC* colorectal cancer

## Conclusions

Fibroblasts have been ignored over decades despite their abundance in the tumor stroma. The pivotal role of CAFs has now emerged in the fields of cancer biology and achieved wide attention. Obviously targeting CAFs or their secretome provides us an effective way to overcome cancers by reducing the immunosuppressive events and remodeling TME but not killing cancer cells directly. Thus, the checkpoint blockade immunotherapies, together with the development of CAF-targeted therapies, hold promise for the treatment of a prevalent tumor that thrives in a fibroblast-rich environment.

However, several challenges must be overcome in order to expedite the leap from bench to bedside. First, the original sources of CAFs in different cancer types remain elusive. Second, due to the original and functional heterogeneity of CAFs, which CAF subtypes populate the immunosuppression TME? Thirdly, are CAF subtypes with distinct phenotypes and immune functions originated from different cellular sources? Finally, the concept that CAF-specific secretome regulates anti-tumor immune response primarily bases on in vitro studies. Therefore, to help accelerate the integration of CAF study into clinical care, future genetic fate mapping and single-cell transcriptional analysis are encourage, which could offer new insights into the heterogeneity, hierarchy, and plasticity of CAFs. Last but not least, we should also carefully consider the in vivo models in order to precisely characterize the function of CAF-released factors that modulate tumor immunity.

## Data Availability

All data generated during this study are included in this published article.

## Abbreviations

APC: Antigen-presenting cell
Arg: Arginase
CAFs: Cancer-associated fibroblasts
CAR: Chimeric antigen receptor
CCL2: Chemokine ligand 2
CXCL12: CX-chemokine ligand 12
CXCL14: CX-chemokine ligand 14
DC: Dendritic cell
ECM: Extracellular matrix
EMT: Epithelial-to-mesenchymal transition
EndMT: Endothelial-to-mesenchymal transition
FAP: Fibroblast activation protein
FASL: FAS ligand
IDO: Indoleamine-2,3-dioxygenase
IL-13: Interleukin-13
IL-6: Interleukin-6
IL-8: Interleukin-8
M-CSF: Macrophage colony-stimulating factor
MDSC: Myeloid-derived suppressor cell
MMPs: Matrix metalloproteinases
MSCs: Mesenchymal stem cells
NK: Natural killer cells
PDGFR: Platelet-derived growth factor
PD-L1/2: Programmed cell death 1 ligand 1/2
PDPN: Podoplanin
S100A4: Fibroblast-specific protein-1
TAMs: Tumor-associated macrophages
TGF-β: Transforming growth factor-β
T_H_ cells: CD4^+^ helper lymphocytes
TME: Tumor microenvironment
Tregs: Regulatory T cell
VEGF: Vascular endothelial growth factor A
α-SMA: α-Smooth muscle actin
